# Effectiveness and moderators of a multicomponent school-based intervention on screen time devices: the *Movimente* cluster-randomized controlled trial

**DOI:** 10.1186/s12889-021-11895-2

**Published:** 2021-10-13

**Authors:** Priscila Cristina dos Santos, Jo Salmon, Lauren Arundell, Marcus Vinicius Veber Lopes, Kelly Samara Silva

**Affiliations:** 1 Federal University of Santa Catarina - Sports center - Physical Education Department, University campus, Trindade, Florianópolis, SC 88040-900 Brazil; 2 Institute for Physical Activity and Nutrition (IPAN), School of Exercise and Nutrition Sciences, Deakin University, 221 Burwood Hwy, Burwood, VIC 3125 Australia

**Keywords:** Recreational screen time, Stationary screen time, Clinical trial, School health, Adolescent

## Abstract

**Background:**

Interventions targeting reduce screen time in adolescents are urgently needed, mainly in low and middle-income countries because of the lack of evidence. Thus, the aims of the study were to examine the effect of a cluster-randomized controlled trial on screen time (ST) devices among Brazilian adolescents and to identify possible moderators.

**Methods:**

Movimente was a multicomponent school-based intervention that was performed in 2017 and consisted of teacher training, education curriculum, and environmental improvements. Baseline and post-intervention assessments (over one academic year) were conducted with students aged 10–16 years at baseline (baseline *n* = 921, [*n* = 538 intervention group; *n* = 383 control group]). A self-report questionnaire was used to measure daily minutes of device specific screen time (TV, computer, video games and smartphone) and demographic variables. Linear mixed models were used to examine intervention effects and an exploratory moderation analysis (sex, grade and socioeconomic status) was performed.

**Results:**

The intervention had no significant effects on TV time (β = − 6.4, 95% CI: − 6.1;13.4), game time (β = − 8.2, 95% CI: − 7.2;10.8), computer time (β = 1.1, 95% CI: − 6.3;18.5), smartphone time (β = − 10.2, 95% CI: − 32.5;12.1), screen time (β = − 12.8, 95% CI: − 50.5;24.8), meeting screen time guidelines (OR: 1.29, 95% CI: 0.65,2.57) and meeting screen time guidelines with smartphone (OR: 1.66, 95% CI: 0.37,7.40). There was a significant intervention effect on reducing TV time (β = − 37.1, 95% CI: − 73.0, − 1.3) among 8th grade students only.

**Conclusions:**

The Movimente intervention was effective only for TV time among 8th grade students. Understanding how school-based interventions can improve adolescents’ device specific screen time across age groups is needed. Future strategies should cover all screen-based devices. Further, there is a need for more studies in low- and-middle income countries to assist in the development of effective strategies.

**Trial registration:**

Clinicaltrials.gov identifier NCT02944318 (25/10/2016).

**Supplementary Information:**

The online version contains supplementary material available at 10.1186/s12889-021-11895-2.

## Introduction

Sedentary behavior is a highly predominant risk behavior adolescents perform throughout the day [1, 2]. Among the different types of sedentary behavior, screen time (ST) is widely investigated and is defined as the time spent on screen-based behaviors performed predominantly in a stationary position, such as watching television (TV) and using computer, video game or smartphones [3]. Evidence shows that exposure of adolescents to excessive ST is associated with several health risks such as increased cardiovascular risk indicators [4], anxiety [5], depression [5] and low social connectedness [6]. International guidelines recommend that children and adolescents should spend less than 2 h per day in recreational ST [7], however, most do not meet this recommendation worldwide [1]. Thus interventions have been encouraged and developed focusing on the reduction of ST in this population group [8–11], with strategies mainly delivered through school environments [12]. The schools are considered promising environments for changing adolescent behavior, it is the place where they spend a large proportion of their daily time and a context conducive to new learning [10, 13].

Although some interventions show significant effects on the reduction of ST [8, 10], systematic reviews highlight that the effect size of interventions are small (highest effect size reported = − 0.29, 95% CI: − 0.35; − 0.22)[14], the methodological quality low [15, 16], and the results are often inconsistent (studies that present conflicting results) [15]. Another aspect is that interventions frequently report the effect on total ST [11, 17]. According to a recent systematic review, most interventions performed in Latin America analysed the intervention effect on clustered computer/videogame and total screen time [18]. However, analysing each screen-based device separately is important to identify whether the intervention’s strategies have device specific effects [8, 9] as they may impact adolescents’ health differently [19]. Excessive TV viewing, for example, is associated with unhealthy diet behaviors and obesity [20], while playing computer/videogames and the use of smartphones is related with unfavourable social behaviors [6] and depression [5]. There is currently little evidence of the impact of interventions on more recent mobile devices such as smartphones, despite being widely used by adolescents [9, 21].

There is also a lack of evidence amongst low and middle-income countries [22]. Although a systematic review of 49 screen time interventions considered in the inclusion criteria studies from high and middle-income countries, all studies included were from high-income countries [21]. In another systematic review of sedentary behavior interventions developed in Latin America, a region composed mainly of low and middle-income countries, only nine studies were included [18]. Screen time behaviors and their correlates differ between high and middle-middle income countries highlighting the need for specific intervention strategies [23].

Furthermore, there is a gap in the literature about which subgroups respond best to interventions to improve adolescent’s health behaviors [24]. Despite this need, the analysis of subgroups has rarely been performed in interventions to reduce ST [9, 16]. Some findings indicate that sex [9], age [8], and socioeconomic status (SES) [25] may influence different responses in interventions. For instance, boys appear to show greater effects and significant reductions in ST compared to girls [9, 26], and younger adolescents (11–13 years old) respond better than older adolescents (14–17 years old) [8]. Considering SES, the higher the income the more time is spent in ST among adolescents in low-middle-income countries, thus SES may be a potential moderator of the intervention effect [23]. Therefore, the aim of this study was to determine the effects of the *Movimente* cluster-randomized controlled trial intervention on ST device use and identify possible moderators (i.e., sex, grade, SES) in adolescents from public schools in Florianopolis, Santa Catarina, Brazil.

## Methods

### Participants and research setting

The *Movimente* Program was a cluster-randomized controlled trial registered on Clinical Trials (NCT02944318–25/10/2016) and approved by the ethics committee of Federal University of Santa Catarina (protocol number: 1.259.910). Written informed consent was obtained from each participant and parents before data collection. The research was performed in accordance with the Declaration of Helsinki.

The target population included 7th to 9th grade students of public municipal schools in Florianopolis, Brazil. The sampling calculations used a statistical power of 80% and a significance level of 5%. It was estimated a minimum sample of 517 subjects (1,1 between intervention and control groups) was required to determine change in physical activity (PA). Considering the sample design (randomized controlled trial - RCT) and the clustered nature of the sampling frame, the sample was doubled to 1,034 students.

Schools with at least two classes in the 7th to 9th grades and not undergoing environment reform (i.e., the school could not be going through a civil construction process) were considered eligible (*n* = 18). An invitation to participate in the *Movimente* program was sent to school principals and seven schools agreed to be part of the program. One school was allocated to the pilot study (conducted prior to the RCT), and three schools were randomized to the control and intervention group respectively. The allocation of schools was matched to ensure a peer-group ratio of 1:1 according to the number of 7th to 9th grade classes and geographic location. All students in the 7th to 9th grades from the six schools who were present in the first 2 weeks of class (1,427 students) were considered eligible. Of these, 921 participated in the study. Further details of the development of the *Movimente* intervention have been described elsewhere [27].

### Intervention Framework

*Movimente* was one academic-year multicomponent intervention with a primary focus on reducing sedentary behavior and promoting physical activity among adolescents. The intervention was based on Social Cognitive Theory [28], Socioecological Frameworks [29] as well as Health Promoting School framework [12] and included three strategy components (teacher training, education curriculum and school environment) (Table 1). The face-to-face teacher training was conducted by the research team after baseline data collection and health topics such as sedentary behavior, physical activity and healthy eating were addressed. The following topics on sedentary behavior were included: sedentary behavior concept; types of sedentary behavior; ST recommendations for children and adolescents; prevalence of excessive sedentary behavior in Brazil and other countries; presentation of a handbook; and discussion of how this handbook information could be used during classes. The handbook was prepared by the program researchers and contained information on different topics and activities for reducing sedentary behavior in and out of school, including class-time activity breaks and motivational messages to reduce ST outside of school. The materials used in the intervention, such as handbooks and educational materials, are available online at http://movimente.ufsc.br/en/.

**Table 1:** The specific sedentary behaviour content delivered in the *Movimente* intervention

Intervention component	Receptor agent	Actions Description	Delivery moments
Teacher training	- General teachers^a^ - Physical education teachers	• Topics of teacher training: Prevalence of sedentary behaviour among adolescents; excessive sedentary behaviour and harms to health; sedentary behaviour concept (i.e., sitting time and ST), ST guidelines and possible activities to perform with students to reduce sedentary behaviour. • Topics of educational material delivered: Suggestions of sedentary behaviour breaks at class room; how to approach the theme of sedentary behaviour in all school subjects. • Online support (i.e., email and social networks during all academic year).	- Soon after baseline data collection, at the beginning of the year;
Educational curriculum	- School managers and teachers	• Delivery of banners and folder: Screen time messages (e.g. excessive ST is bad for your health; try to reduce your ST and spend more time with your family and friends.). • It was suggested to teachers and coordinators to performed activities with students in order to show the folders to parents or guardians to disseminate this information.	- The folders were delivered every two months; - The banners were delivered at the beginning and middle of the year.
School environment^b^	- Out of classroom spaces at school	• Revitalization of old courts;	- The environmental change occurred at the beginning of the year.

*Note*. ^a^General teacher: Teachers of all disciplines, excluding physical education (e.g. Math, History, Geography etc.), *ST* screen time. ^b^Direct actions for physical activity with a focus on replacing sedentary behaviour are best described in the protocol paper of the *Movimente* school-based program

Two teacher training sessions were carried out for i) physical education teachers and ii) general teachers (e.g. Math, Geography, Biology). Both trainings were similar, however, there was less theoretical content and more discussion about pedagogical practice with physical education teachers. In addition, the handbooks were specific for each grade only for Physical Education (i.e. one handbook was prepared for the 7th grade, another for the 8th grade and another for the 9th grade). After the training session, the research team offered online support during the academic year by email and social networks, to assist teachers in the application of the contents proposed during teacher training.

The *Movimente* educational curriculum component consisted of banners and folders containing content about sedentary behavior, physical activity, healthy eating and the relationship between physical activity and academic achievement. The four banners were handed over to the school coordination at the beginning of the year, and program researchers advised the school staff to make these posters available in strategic locations to reach as many students as possible. Folders with educational content were also delivered to the schools every 2 months (every 2 months a type of content). Teachers were encouraged to discuss the contents of these folders with students and to perform activities involving parents to disseminate information to the students’ families.

Finally, the revitalization of sports courts was performed, as well as the creation of new spaces in order to provide greater opportunities for physical activity and reduce sedentary behavior among students. Sports equipment (i.e., balls, rackets and ropes) were delivered to the school coordination to be used by the students during recess and periods without class. It was up to the school to organize the availability of this material. Physical education teachers were encouraged to use the created spaces. Banners informing students of the sporting materials were also provided to schools. All the delivered materials were managed and used by the school staff, without interference from the program researchers. Detailed information about the intervention have been described elsewhere [27].

### Measurements

Participants completed a self-report survey at school. ST was measured via questions based on the Youth Risk Behavior Survey Questionnaire [30], validated for the Brazilian population [31] and extended for each screen device separately for weekdays and weekends (e.g., Generally, how many hours of television do you watch on a weekday/weekend day?). The same questions were asked of: use computer/video game for gaming purpose; use the computer, except for gaming use and use smartphone. For each ST device question there were eight answer options (I do not watch/play/use; Less than 1 h a day; 1 h a day; 2 h a day; 3 h a day; 4 h a day; 5 h a day and 6 or more hours a day), which were transformed into a linear scale of values ranging from zero to six (I do not watch/play/use = 0; Less than 1 h a day = 0.5; 1 h = 1; 2 h = 2, and so on) and weighted according to weekdays and weekend days by applying the following equation: [(Screen device use minutes/week * 5) + (Screen device use minutes/weekend * 2) / 7]. The ST variable was obtained by summing the time of use of TV, game and computer, but not smartphone as applied previously [3]. However, a second ST variable which includes smartphone [9] was also considered in the present study (i.e., obtained by summing the time of use of TV, game, computer and smartphone). The intervention effect on ST recommendation was analysed considering the cut-off point of less than 2 h per day [3].

Participants also reported their sex, age, grade and the quantity of household items listed on a checklist based on the Brazilian Economic Classification Criteria (e.g. number of televisions, refrigerators, microwaves, cars). An asset index was calculated using Principal Component Analysis on the list of 16 household items. The index was used as a proxy of SES and was categorized according to tertiles. Higher terciles in an increasing order can be interpreted as greater family wealth.

### Procedures

The intervention was conducted over one academic year in 2017. In Brazil, the academic year of municipal schools starts between February and early March, and ends in December. Thus, the intervention was implemented over the academic year and not the complete year, which excludes the period without regular classes (vacations, usually in July). In the first weeks of the school years (last week of February), we contacted the school principals and organized the data collection which started in March. The data collection process was performed in March (pre-intervention) and December (post intervention) by trained and calibrated researchers to standardize the entire procedure, regardless of whether data collection was performed in the control or intervention group. Schools allocations were performed prior to baseline data collection. Although the researchers knew which schools were in each group (control and intervention), training was provided so that they did not influence data collection. The data were tabulated using an optical reading software “Sphinx Survey”.

### Statistical analysis

Statistical analyses were conducted using Stata 16.0 (StataCorp LP., College Station, TX, USA). Mean and standard deviation were calculated for continuous variables; prevalence and 95% confidence interval were calculated for categorical variables. Wilcoxon and Pearson’s chi-square tests were used to compare treatment (control vs intervention) at baseline and participants with dropouts.

Due to the use of school as a randomization unit and the structure of the data, three-level linear and logistic mixed models were performed for continuous and categorical outcomes, respectively. The following hierarchical structures were applied to all models: repeated measures (level 1), subjects (level 2), and schools (level 3). As mixed models can accommodate unbalanced data, all available measures were included in analysis. Base models were created by including indicator variables for allocation group (control or intervention), time (baseline or follow-up) and its interaction term (group*time) as fixed effects. The variables sex, grade and SES were then included as adjustments among the fixed effects. An exploratory moderation analysis was undertaken by including three-way interaction terms between group, time and the adjustment variables (group*time*moderator). Simple main effects were explored for the three-way interaction terms significant at *p*-value < 0.10 [32].

Fitted models were evaluated according to the assumptions of homoscedasticity and residuals normality. Due to slightly skewed residuals observed in some models, a bootstrapping procedure was conducted to obtain corrected standard errors (1500 resamples). Results of ST changes from pre- to post-intervention were expressed as mean differences (β) for continuous outcomes and Odds Ratio (OR) for categorical outcomes. Standardized effect sizes (stdβ) were computed for group differences of pre- to post-intervention changes.

## Results

**Fig. 1:**
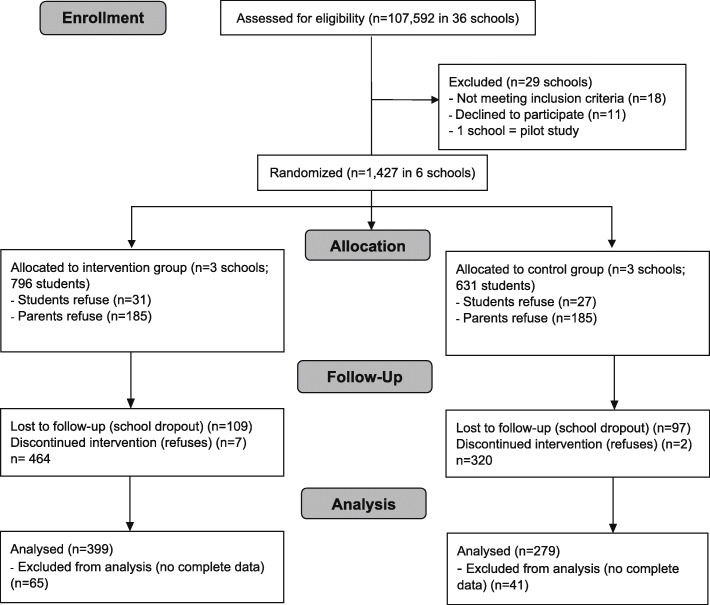
CONSORT flow diagram of screen time in the *Movimente* study

Of the 1427 eligible students from the six schools, 921 students answered the questionnaire at baseline (*n* = 538 intervention group; *n* = 383 control group; response rate: 65%) and 788 participants provided complete baseline data (*n* = 472 intervention group, *n* = 316 control group). From those, 678 provided complete post-intervention data (*n* = 399 intervention group, *n* = 279 control group) (Fig. 1).

Table 2 shows the baseline characteristics of students and the time spent per day on different screen devices. There were no significant differences in sociodemographic variables and ST duration between participants and dropouts (Additional file 1: Table A1). No differences were found between the control and intervention groups at baseline for any demographic or ST variables, except for game time (*p*-value = 0.013) as shown in Table 2. Adolescents spent an average of 299 min per day in ST, and most adolescents did not meet the ST recommendation (97.2%).

**Table 2:** Baseline characteristics of the *Movimente* study sample

	Control (***n*** = 316)	Intervention (***n*** = 472)	
Variables	mean (SD) or n (%)	mean (SD) or n (%)	***p***-value
Sex			0.582
Male	145 (45.9)	226 (47.9)	
Female	171 (54.1)	246 (52.1)	
Age (years)	13.13 ± 1.07	13.05 ± 1.02	
Grade			0.833
7th grade	117 (37.0)	169 (35.8)	
8th grade	96 (30.4)	153 (32.4)	
9th grade	103 (32.6)	150 (31.8)	
SES			0.100
1st tertile	121 (38.3)	146 (30.9)	
2nd tertile	96 (30.4)	163 (34.5)	
3th tertile	99 (31.3)	163 (34.5)	
Time per day (min)			
TV	148.07 ± 111.24	145.75 ± 102.33	0.763
Games	84.90 ± 115.85	110.30 ± 120.81	0.003
Computer	66.04 ± 88.52	77.21 ± 96.12	0.099
Smartphone	202.68 ± 128.17	217.75 ± 123.25	0.098
Screen time	299.01 ± 207.24	333.26 ± 217.39	0.028
Screen time w/ smartphone	501.69 ± 255.99	551.00 ± 260.83	0.009
ST guidelines			0.135
≥ 2 h	252 (79.7)	396 (83.9)	
< 2 h	64 (20.3)	76 (16.1)	
ST guidelines w/ smartphone			0.938
≥ 2 h	307 (97.2)	459 (97.2)	
< 2 h	9 (2.8)	13 (2.8)	

*Note*: *Min* minutes, *SD* standard deviation, *SES* socioeconomic status, *ST* screen time, * represent significant difference between treatments (control vs. intervention)

Table 3 shows the intervention effect on ST devices. According to the group-by-time interaction analysis there was no significant effect of the intervention condition after adjusting for sex, grade and SES in TV time (β = − 6.4, 95% CI: − 6.1;13.4), game time (β = − 8.2, 95% CI: − 7.2;10.8), computer time (β = 1.1, 95% CI: − 6.3;18.5), smartphone time (β = − 10.2, 95% CI: − 32.5;12.1), ST (β = − 12.8, 95% CI: − 50.5;24.8), ST guidelines (OR: 1.29, 95% CI: 0.65,2.57) and ST guidelines with smartphone (OR:1.66, 95% CI: 0.37,7.40). Thus, no differences in changes in ST device use over time between control and intervention conditions were observed.

**Table 3:** Effect of *Movimente* intervention on different screen time devices in adolescents

Outcomes	Time effect for the control group	Time effect for the intervention group	Intervention vs control time effect contrast		
	β (95% CI)	β (95%CI)	β (95% CI)	std β	***p***-value
TV time (min/day)	−7.6 (− 22.8;7.6)	− 14.0 (− 26.4; − 1.5)	−6.7 (− 6.1;13.4)	− 0.06	0.528
Game time (min/day)	−2.6 (− 17.8;12.6)	− 10.8 (− 22.7;1.1)	−8.2 (− 7.2;10.8)	−0.07	0.397
Computer time (min/day)	−2.8 (− 14.9;9.2)	−1.7 (− 14.0;10.6)	1.13 (− 6.3;18.5)	0.01	0.898
Smartphone time (min/day)	17.7 (−0.4;35.8)	7.5 (− 6.1;21.0)	− 10.2 (− 32.5;12.1)	− 0.08	0.371
ST (min/day)	−13.5 (− 1.8;14.8)	− 26.4 (− 50.6;-2.1)	−12.8 (− 50.5;24.8)	−0.06	0.504
ST w/ smartphone (min/day)	4.3 (−30.3; 38.9)	− 18.9 (− 46.5; 8.8)	−23.2 (− 67.9;21.5)	− 0.09	0.309
ST guidelines (<2hs)^a^	1.48 (0.89;2.48)	1.92 (1.20;3.06)	1.29 (0.65;2.57)	–	0.464
ST guidelines w/ smartphone (<2hs)^a^	0.88 (0.27;2.84)	1.46 (0.58;3.69)	1.66 (0.37;7.40)	–	0.507

*Note*. Data presented as the slope of time (post- vs pre- intervention) from the fixed part of the model, *ST* screen time; ^a^ Expressed as OR for meeting recreational screen time guidelines (< 2 h)

Possible differences in intervention effects between subgroups were analysed by examining the interactions effects for sex, grade and SES. The three-way interaction terms (group*time*moderator) showed no differences in the effects of the intervention between sex, grade and SES for game time, computer time, smartphone time, ST, ST guidelines and ST guidelines with smartphone (Additional file 1: Tables A2, A3 and A4). However, an interaction effect of grade on time by group term for TV (*p*-value = 0.089) was observed. There was an intervention effect on reducing TV time (β = − 37.1, 95% CI: − 73.0, − 1.3) only among 8th grade students (Table 4).

**Table 4:** Effect of *Movimente* intervention on different screen time devices according to school grade among adolescents

Outcomes	Time effect for the control group	Time effect for the intervention group	Intervention vs control time effect contrast		
	β (95% CI)	β (95% CI)	β (95% CI)	std β	***p***-value
**TV time (min/day)**					
7th grade	−1.5 (−26.9; 23.9)	2.7 (− 17.4; 22.9)	4.2 (− 28.3; 36.8)	0.04	0.800
8th grade	6.8 (−22.1; 35.7)	−30.3 (−51.6; −9.1)	−37.1 (− 73.0; − 1.3)	− 0.35	0.043*
9th grade	−32.1 (− 58.9; − 5.2)	− 15.8 (− 39.0; 7.4)	16.3 (− 18.5; 51.0)	0.15	0.359

*Note*. Data presented as the slope of time (post- vs pre- intervention) from the fixed part of the model. * represents significant difference of changes between treatments (control vs. intervention)

## Discussion

This study examined whether a multicomponent intervention was effective in reducing overall ST and device specific ST among adolescents. There was no effect of the *Movimente* intervention on each screen device among the overall sample. However, strategies were effective in reducing TV viewing of adolescents in 8th grade by almost 40 min per week. Despite evidence that TV viewing time is being replaced by other new screen devices [33], TV still represents one of the most accessed devices in this population group, particularly in low- and middle-income countries [34] and among people of low SES [33]. According to the latest nationwide survey conducted in Brazil, 60% of 9th grade students watch more than 2 h of TV per day [35] and a systematic review showed that 58.8% of Brazilian adolescents (10–19 years) had excessive TV time. Given consistent associations between TV viewing and unhealthy outcomes [33, 34], this reduction in TV viewing may have a beneficial impact on these adolescents’ health.

The effect found only for the 8th grade may be explained by the age of the students, the characteristics of elementary schools in Brazil and strategies of the *Movimente* study. Perhaps the ST behavior of younger students (7th grade) is more dependent and influenced by parents and the *Movimente* intervention had no specific strategies either for the parents or relatives, or for the family environment. Interventions with family involvement are often more effective, particularly for reducing recreational ST [14, 36]. In comparison, 9th grade students have many different school activities, the school calendar ends earlier and they are preparing for high school, which may have made participation and access to *Movimente* program strategies even more difficult. Thus, the results suggest that it may be interesting to consider the different characteristics of the grades in the formulation of intervention strategies. In addition, participants’ perception of changes to their screen time throughout the intervention was not evaluated but may be interesting in further investigations.

Although the effect of the Movimente intervention on ST was not gender-moderated, other studies have shown that gender can moderate intervention effects [8, 37]. A survey study amongst French students showed that recreational ST is strongly sex dependent, with boys participating more in games (play video games with consoles, tablets, smartphones, or other electronic media) and girls in social media interactions [38]. Even in intervention studies with all participants undergoing the same strategies, there is a reduction in TV time among girls but not among boys [8] and a reduction in computer time only in boys [39]. To develop policies and actions that are more inclusive, future interventions need to document gender differences and similarities in strategies and results, since information reported in the literature is not enough yet [37].

Most studies that have investigated the effect of interventions on reducing ST among adolescents, have found distinct results for different screen components. For example, while one study observed a reduction for TV viewing and total ST [40], another study found it for TV viewing and computer/video game time [8], and another show it for computer time [9]. It is possible that some ST behaviors are more difficult to reduce or need specific intervention strategies. For example, the main strategies of the ACTIVITAL intervention focused on TV, which resulted in a greater reduction in the time spent watching TV but to the detriment of other ST behaviors [40]. Therefore, it seems that the intervention strategies need to be better elaborated to cover all screen-based devices as the excessive recreational ST provides harmful effects on health. Strategies need to be tailored towards specific behaviors (i.e. TV viewing and/or computer time), as trying to change multiple screen behaviors without specific strategies, i.e. the “one fits for all” approach, does not seem to be effective.

In addition, many interventions neglect new technologies such as smartphones, which are widely used by adolescents [3, 21]. Our intervention was not effective at reducing smartphone time. Only two other interventions with adolescents have examined smartphone use [9, 11], however, both studies included smartphone use in their sum of overall ST measure. This makes understanding and comparability difficult where other studies consider ST as TV, computer and video game time [3]. One of these interventions did also consider the smartphone separately and observed significant reduction in the prevalence of adolescents that do not meet recommendations (≥2 h per day) after intervention [9]. The study suggests that curricular and extracurricular actions, and greater involvement of students, the school community and family members are effective strategies in reducing smartphone use in adolescents [9].

The lack of effectiveness for the whole sample may be due to the strategies not being focused exclusively on ST since the *Movimente* intervention also focused on physical activity and other health behaviors [15]. Similarly, an intervention performed with students from New South Wales (Australia) that focused on several health behaviors (BMI, physical activity, emotional problems, behavioral problems and psychological well-being) and applied strategies similar to the current RCT but with more family involvement, health messaging and parental newsletters, also found no effect in reducing ST (− 21.3 min/day; *p* = 0.255, 11]. There was also no effect of a multicomponent intervention focused on promoting physical activity and healthy eating habits performed with Brazilian adolescents [41].

In contrast, a cluster RCT performed with adolescents in Cuenca (Ecuador) found significant effects for TV viewing (− 15.7 min/day; *p* = 0.003) and total ST (− 25.9 min/day; *p* = 0.03) after 18-months [40] with a multicomponent intervention. However, when the intervention strategies focusing on reducing ST stopped and the intervention focus changed to diet and physical activity, the effects were not maintained [40]. According to a recent systematic review, some interventions with high methodological quality that focused exclusively on ST reduction showed intervention effectiveness [15]. An umbrella systematic review found that interventions with strategies focused exclusively on sedentary behavior were more effective in reducing ST and sitting time than those with strategies towards both physical activity and sedentary behavior combined [22]. Though there is still no consistent evidence [15, 16], the results of these studies suggest that the interventions may be more effective if focused exclusively on reducing ST [15]. It is also important to highlight that interventions with a multicomponent approach have been widely used [14] and present an important design to promote behavioral change due to focusing on interrelated determinants of one or codependent outcomes. However, the interpretation of effects is limited to the whole intervention as the effectiveness of isolated strategies cannot be precisely estimated. Thus, qualitative process evaluation should be designed within school-based interventions to expand the understanding of the implementation’s reach and magnitude.

The lack of significant effects in the current study may also relate to intervention implementation. Poor implementation is one of main reasons that ST interventions have been unsuccessful [42]. For instance, our intervention reached only 19 of the 63 teachers (30%) who could have participated in the teacher training. After the intervention, teachers were asked which topics covered during teacher training (i.e., physical activity, diet, physical activity related to academic performance, and ST) were used in their class and ST was the least addressed. This was also identified by the students who, when asked about the topics covered during the classes, recalled more topics about physical activity and diet, with ST being rarely addressed. Thus, low teacher participation and students’ recall of the topics covered during the intervention may have contributed to the lack of effect [43].

Further, many of the strategies developed in the *Movimente* intervention were based on interventions performed in high-income countries [17]. The context of these countries is completely different from low- and middle-income countries, which may also affect implementation and effectiveness. For example, Australia [17], United States [10] and Finland [44] that develop good intervention models are highly ranked in regard to the educational system, while Brazil is in the last positions, with a school context where teachers are devalued and government incentives for public education are lacking. Another important difference is that most Brazilian schools are predominantly part-time, with students spending 4 h daily at school in a morning or afternoon ‘shift’. This means they are likely to be less exposed to the actions of the intervention than students in countries that require them to spend six or more hours at school at least5 days per week. There is a need for further studies to evaluate the implementation of intervention strategies in low- and middle-income countries.

This study has some limitations such as the ST variable measured by self-report, however, the used instrument has the strength of comprising multiple screen devices and was previously validated with this population [31]. The retention and response rates (65 and 80%, respectively) were lower than expected. Thus, sensitivity analyses of subgroups were conducted and no evidence of selection bias was observed. We understand that the effects on physical activity and screen time cannot be separated and are expected to be dependent due to time displacement. As an example, if strategies towards reducing overall screen time were successful, students would need to fill their extra time with another behavior. Physical activity may be the behavior of choice as stimulated by the intervention strategies focused on promoting this behavior. In such as case we cannot assume that changes of screen time were secondary or residual to our intervention. Another limitation was the low participation of teachers in teacher training, and the students’ lack of recall of the topics covered during the intervention.

The major strengths of this study are the randomized controlled trial design and the use of multilevel analysis, an approach that allowed to consider the nested nature of the data which are important to reduce inference bias and to avoid misleading conclusions on population estimates. It is also important to highlight the development of this intervention in a middle-income country that needs more evidence to support the formulation and implementation of future interventions more effective and better implemented.

## Conclusion

No differences between intervention and control groups on overall or device-specific ST were observed in the whole sample of the *Movimente* study. However, moderation analysis showed that strategies were effective for reducing TV time among 8th grade students. No other interaction effects were observed. Future interventions may be more effective if they only focus on reducing screen time instead of focusing on multiple health behaviors and with more family engagement. In addition, there is a gap in knowledge regarding which strategies are most effective for reducing ST, thus mediation analyses are needed to advance this area. More studies on the effectiveness and implementation of interventions in low- and middle-income countries are also needed to better understand the intervention process in different contexts.

## Supplementary Information

**Additional file 1: Table A1.** Comparison between participants and dropouts. Note: Min: minutes; SES: socioeconomic status; *p*-value represents the results of t-teste and Chi-square test comparing participants and dropouts. **Table A2.** Effect of *Movimente* intervention on different screen time devices according to grade among adolescents. Note: Data presented as the slope of time (post- vs pre- intervention) from the fixed part of the model. **Table A3.** Effect of *Movimente* intervention on different screen time devices according to sex among adolescents. Note: Data presented as the slope of time (post- vs pre- intervention) from the fixed part of the model. **Table A4.** Effect of *Movimente* intervention on different screen time devices according to SES tertiles among adolescents. Note: Data presented as the slope of time (post- vs pre- intervention) from the fixed part of the model.

## Data Availability

The dataset and protocol supporting the conclusions of this article are available on request of the corresponding author. All materials (questionnaires) used for the purpose of this study are available on request of the corresponding author.

## Abbreviations

RCT: Randomized controlled trial
ST: Screen time
SES: Socioeconomic status
TV: Television
