# Supplementary material for: Effectiveness and moderators of a multicomponent school-based intervention on screen time devices: the Movimente cluster-randomized controlled trial
Source: BMC Public Health. 2021 Oct 13;21:1852. doi: 10.1186/s12889-021-11895-2 (PMC8515678; doi:10.1186/s12889-021-11895-2)
Supplement: Supplementary file 1 — Additional file 1: Table A1. Comparison between participants and dropouts. Note: Min: minutes; SES: socioeconomic status; p-value represents the results of t-teste and Chi-square test comparing participants and dropouts. Table A2. Effect of Movimente intervention on different screen time devices according to grade among adolescents. Note: Data presented as the slope of time (post- vs pre- intervention) from the fixed part of the model. Table A3. Effect of Movimente intervention on different screen time devices according to sex among adolescents. Note: Data presented as the slope of time (post- vs pre- intervention) from the fixed part of the model. Table A4. Effect of Movimente intervention on different screen time devices according to SES tertiles among adolescents. Note: Data presented as the slope of time (post- vs pre- intervention) from the fixed part of the model. [file 12889_2021_11895_MOESM1_ESM.docx]

**ADDITIONAL FILE**

**Table A1.** Comparison between participants and dropouts.

|  |  | Participants |  | Dropouts |  |
| --- | --- | --- | --- | --- | --- |
| Variables | n | Proportion or mean | n | Proportion or mean | p-value |
| **Sex** |  |  |  |  | 0.700 |
| Male | 357 | 48.6 | 88 | 47.1 |  |
| Female | 377 | 51.4 | 99 | 52.9 |  |
| **Grade** |  |  |  |  | 0,324 |
| 7^th^ grade | 275 | 37.7 | 62 | 33.3 |  |
| 8^th^ grade | 236 | 32.3 | 58 | 31.1 |  |
| 9^th^ grade | 219 | 30.0 | 66 | 35.5 |  |
| **SES** |  |  |  |  | 0.753 |
| 1^st^ tertile | 126 |  | 152 |  |  |
| 2^nd^ tertile | 103 |  | 172 |  |  |
| 3^th^ tertile | 107 |  | 166 |  |  |
| **Time per day (min):** |  |  |  |  |  |
| TV | 723 | 138.7 | 183 | 148.8 | 0.314 |
| Games | 727 | 95.0 | 185 | 98.8 | 0.894 |
| Computer | 719 | 68.4 | 183 | 54.6 | 0.157 |
| Smartphone | 712 | 207.3 | 182 | 220.8 | 0.201 |
| Screen time | 707 | 303.5 | 180 | 304.1 | 0.833 |
| **Screen time guidelines** |  |  |  |  | 0.797 |
| < 2 hours | 147 | 79.2 | 39 | 78.3 |  |
| ≥ 2 hours | 560 | 20.8 | 141 | 21.7 |  |

Note: Min: minutes; SES: socioeconomic status; p-value represents the results of t-teste and Chi-square test comparing participants and dropouts.

**Table A2.** Effect of Movimente intervention on different screen time devices according to grade among adolescents.

| **Outcomes** | **Time effect for the  control group** | **Time effect for the  intervention group** | **Intervention vs control time effect contrast** | | |
| --- | --- | --- | --- | --- | --- |
|  | **β (95%CI)** | **β (95%CI)** | **β (95%CI)** | **std β** | **p-value** |
| **TV time (min)** |  |  |  |  |  |
| 7^th^ grade | -1.47 (-26.87,23.93) | 2.74 (-17.37,22.85) | 4.21 (-28.34,36.76) | 0.04 | 0.800 |
| 8^th^ grade | 6.80 (-22.08,35.68) | **-30.34 (-51.61,-9.07)** | **-37.14 (-73.02,-1.25)** | -0.35 | 0.043 |
| 9^th^ grade | **-32.05 (-58.86,-5.23)** | -15.79 (-39.03,7.44) | 16.26 (-18.49,51.00) | 0.15 | 0.359 |
| **Videogame time (min)** |  |  |  |  |  |
| 7^th^ grade | -6.60 (-34.85,21.66) | -3.83 (-22.68,15.03) | 2.77 (-31.07,36.61) | 0.02 | 0.873 |
| 8^th^ grade | -0.59 (-28.94,27.77) | **-28.83 (-50.03,-7.62)** | -28.24 (-63.43,6.95) | -0.24 | 0.116 |
| 9^th^ grade | -0.39 (-17.26,16.47) | 0.52 (-19.86,20.90) | 0.92 (-25.46,27.29) | 0.01 | 0.946 |
| **Computer time (min)** |  |  |  |  |  |
| 7^th^ grade | -10.04 (-30.90,10.82) | 11.17 (-9.05,31.39) | 21.21 (-8.51,50.93) | 0.23 | 0.162 |
| 8^th^ grade | -4.05 (-27.19,19.09) | 1.11 (-20.26,22.48) | 5.16 (-26.58,36.90) | 0.06 | 0.750 |
| 9^th^ grade | 7.46 (-12.70,27.63) | -19.62 (-41.52,2.28) | -27.08 (-57.24,3.07) | -0.29 | 0.078 |
| **Smartphone time (min)** |  |  |  |  |  |
| 7^th^ grade | 18.23 (-13.25,49.71) | 10.56 (-13.64,34.75) | -7.67 (-46.89,31.54) | -0.06 | 0.701 |
| 8^th^ grade | 9.55 (-22.25,41.35) | -4.97 (-28.93,18.99) | -14.52 (-53.89,24.86) | -0.12 | 0.470 |
| 9^th^ grade | 26.30 (-5.42,58.03) | 17.14 (-7.11,41.39) | -9.16 (-49.36,31.03) | -0.07 | 0.655 |
| **Screen time (min)** |  |  |  |  |  |
| 7^th^ grade | -18.26 (-69.72,33.20) | 10.65 (-29.19,50.48) | 28.91 (-36.99,94.80) | 0.13 | 0.390 |
| 8^th^ grade | 2.08 (-48.89,53.04) | **-58.21 (-99.56,-16.85)** | -60.28 (-125.93,5.36) | -0.28 | 0.072 |
| 9^th^ grade | -26.08 (-69.68,17.51) | -35.00 (-78.74,8.75) | -8.91 (-70.65,52.83) | -0.04 | 0.777 |
| **Screen time w/ smartphone (min)** |  |  |  |  |  |
| 7^th^ grade | 1.01 (-67.65,69.67) | 21.51 (-22.58,65.60) | 20.50 (-61.11,102.12) | 0.08 | 0.622 |
| 8^th^ grade | 10.89 (-42.16,63.95) | **-62.95 (-111.70,-14.21)** | **-73.85 (-145.88,-1.82)** | -0.29 | 0.044 |
| 9^th^ grade | 0.30 (-56.32,56.93) | -18.38 (-70.29,33.53) | -18.68 (-95.74,58.37) | -0.07 | 0.635 |

Note: Data presented as the slope of time (post- vs pre- intervention) from the fixed part of the model.

**Table A3.** Effect of Movimente intervention on different screen time devices according to sex among adolescents.

| **Outcomes** | **Time effect for the  control group** | **Time effect for the  intervention group** | **Intervention vs control time effect contrast** | | |
| --- | --- | --- | --- | --- | --- |
|  | **β (95%CI)** | **β (95%CI)** | **β (95%CI)** | **std β** | **p-value** |
| **TV time (min)** |  |  |  |  |  |
| Male | 0.88 (-22.79,24.56) | -3.90 (-20.59,12.79) | -4.78 (-34.25,24.69) | -0.04 | 0.750 |
| Female | -15.04 (-35.52,5.45) | **-23.40 (-41.62,-5.19)** | -8.37 (-35.57,18.83) | -0.08 | 0.546 |
| **Videogame time (min)** |  |  |  |  |  |
| Male | 0.82 (-24.09,25.73) | -5.15 (-23.97,13.67) | -5.97 (-37.13,25.18) | -0.05 | 0.707 |
| Female | -5.49 (-23.51,12.53) | **-16.16 (-30.40,-1.92)** | -10.67 (-33.60,12.26) | -0.09 | 0.362 |
| **Computer time (min)** |  |  |  |  |  |
| Male | -5.33 (-23.47,12.81) | 0.62 (-15.95,17.20) | 5.95 (-18.89,30.79) | 0.06 | 0.639 |
| Female | -0.65 (-16.81,15.51) | -3.90 (-21.76,13.96) | -3.25 (-27.56,21.06) | -0.03 | 0.793 |
| **Smartphone time (min)** |  |  |  |  |  |
| Male | 15.14 (-12.51,42.79) | -0.30 (-19.35,18.76) | -15.43 (-48.45,17.58) | -0.12 | 0.360 |
| Female | 19.88 (-4.31,44.07) | 14.74 (-5.04,34.52) | -5.14 (-36.31,26.03) | -0.04 | 0.746 |
| **Screen time (min)** |  |  |  |  |  |
| Male | -4.12 (-49.84,41.61) | -8.20 (-42.27,25.87) | -4.08 (-61.05,52.88) | -0.02 | 0.888 |
| Female | -21.60 (-57.68,14.48) | **-43.35 (-77.53,-9.18)** | -21.75 (-71.51,28.00) | -0.10 | 0.391 |
| **Screen time w/ smartphone (min)** |  |  |  |  |  |
| Male | 11.15 (-42.73,65.03) | -8.69 (-51.08,33.70) | -19.84 (-88.12,48.44) | -0.08 | 0.569 |
| Female | -1.51 (-48.24,45.23) | -28.50 (-65.60,8.61) | -26.99 (-86.61,32.62) | -0.10 | 0.375 |

Note: Data presented as the slope of time (post- vs pre- intervention) from the fixed part of the model.

**Table A4.** Effect of Movimente intervention on different screen time devices according to SES tertiles among adolescents.

| **Outcomes** | **Time effect for the  control group** | **Time effect for the  intervention group** | **Intervention vs control time effect contrast** | | |
| --- | --- | --- | --- | --- | --- |
|  | **β (95%CI)** | **β (95%CI)** | **β (95%CI)** | **std β (95%CI)** | **p-value** |
| **TV time (min)** |  |  |  |  |  |
| 1st SES tertile | -18.69 (-42.83,5.45) | -14.27 (-37.82,9.27) | 4.42 (-29.38,38.21) | 0.04 | 0.798 |
| 2nd SES tertile | 12.06 (-15.33,39.44) | -0.60 (-19.26,18.06) | -12.65 (-46.27,20.96) | -0.12 | 0.461 |
| 3rd SES tertile (higher SES) | -12.33 (-43.48,18.81) | -27.81 (-50.27,-5.35) | -15.47 (-53.95,23.01) | -0.15 | 0.431 |
| **Videogame time (min)** |  |  |  |  |  |
| 1st SES tertile | -1.59 (-27.72,24.53) | -10.05 (-32.83,12.74) | -8.45 (-42.70,25.79) | -0.07 | 0.628 |
| 2nd SES tertile | -7.51 (-30.09,15.08) | -13.25 (-33.88,7.39) | -5.74 (-35.77,24.29) | -0.05 | 0.708 |
| 3rd SES tertile (higher SES) | 0.92 (-26.97,28.81) | -9.15 (-27.15,8.86) | -10.07 (-42.69,22.55) | -0.08 | 0.545 |
| **Computer time (min)** |  |  |  |  |  |
| 1st SES tertile | -3.02 (-19.98,13.93) | 11.58 (-11.90,35.05) | 14.60 (-13.81,43.01) | 0.16 | 0.314 |
| 2nd SES tertile | 7.58 (-18.17,33.33) | -14.17 (-32.65,4.30) | -21.75 (-53.37,9.86) | -0.23 | 0.177 |
| 3rd SES tertile (higher SES) | -12.39 (-34.22,9.44) | -0.31 (-22.09,21.46) | 12.08 (-19.37,43.52) | 0.13 | 0.452 |
| **Smartphone time (min)** |  |  |  |  |  |
| 1st SES tertile | 7.83 (-23.63,39.28) | 8.75 (-17.49,35.00) | 0.92 (-39.74,41.58) | 0.01 | 0.964 |
| 2nd SES tertile | 22.45 (-11.88,56.78) | 1.54 (-22.59,25.67) | -20.91 (-63.30,21.47) | -0.17 | 0.334 |
| 3rd SES tertile (higher SES) | 25.23 (-4.40,54.87) | 12.75 (-8.71,34.21) | -12.48 (-49.55,24.58) | -0.1 | 0.509 |
| **Screen time (min)** |  |  |  |  |  |
| 1st SES tertile | -23.92 (-68.71,20.87) | -11.65 (-55.68,32.39) | 12.28 (-49.88,74.43) | 0.06 | 0.699 |
| 2nd SES tertile | 11.10 (-39.13,61.33) | -27.49 (-66.06,11.08) | -38.59 (-101.22,24.04) | -0.18 | 0.227 |
| 3rd SES tertile (higher SES) | -23.53 (-78.23,31.18) | -38.26 (-80.75,4.22) | -14.74 (-85.43,55.96) | -0.07 | 0.683 |
| **Screen time w/ smartphone (min)** |  |  |  |  |  |
| 1st SES tertile | -16.00 (-69.12,37.12) | -3.17 (-55.98,49.65) | 12.84 (-60.53,86.20) | 0.05 | 0.732 |
| 2nd SES tertile | 33.61 (-30.21,97.43) | -25.95 (-69.36,17.45) | -59.56 (-136.64,17.52) | -0.23 | 0.130 |
| 3rd SES tertile (higher SES) | 2.19 (-67.32,71.70) | -25.45 (-74.77,23.88) | -27.64 (-114.17,58.89) | -0.11 | 0.531 |

Note: Data presented as the slope of time (post- vs pre- intervention) from the fixed part of the model
